# Maternal high-fat diet consumption impairs exercise performance in offspring

**DOI:** 10.1017/jns.2014.55

**Published:** 2014-12-17

**Authors:** Isabel Walter, Susanne Klaus

**Affiliations:** Group of Energy Metabolism, German Institute of Human Nutrition in Potsdam, Arthur Scheunert Allee 114–116, 14558 Nuthetal, Potsdam-Rehbruecke, Germany

**Keywords:** High-fat diet, Offspring, Training, Skeletal muscle, CD36, cluster of differentiation 36, Cpt1b, carnitine O-palmitoyltransferase 1b, Esrrg, oestrogen-related receptor-γ, eWAT, epididymal white adipose tissue, FAS, fatty acid synthase, HFD, high-fat diet, LFD, low-fat diet, mHFD, maternal high-fat diet, mLFD, maternal low-fat diet, REE, resting energy expenditure, RQ, respiratory quotient, RW, running wheel, Serca2, sarcoplasmic/endoplasmic reticulum Ca ATPase 2

## Abstract

The aim of the present study was to scrutinise the influence of maternal high-fat diet (mHFD) consumption during gestation and lactation on exercise performance and energy metabolism in male mouse offspring. Female C3H/HeJ mice were fed either a semi-synthetic high-fat diet (HFD; 40 % energy from fat) or a low-fat diet (LFD; 10 % energy from fat) throughout gestation and lactation. After weaning, male offspring of both groups received the LFD. At the age of 7·5 weeks half of the maternal LFD (*n* 20) and the mHFD (*n* 21) groups were given access to a running wheel for 28 d as a voluntary exercise training opportunity. We show that mHFD consumption led to a significantly reduced exercise performance (*P* < 0·05) and training efficiency (*P* < 0·05) in male offspring. There were no effects of maternal diet on offspring body weight. Lipid and glucose metabolism was disturbed in mHFD offspring, with altered regulation of cluster of differentiation 36 (CD36) (*P* < 0·001), fatty acid synthase (*P* < 0·05) and GLUT1 (*P* < 0·05) gene expression in skeletal muscle. In conclusion, maternal consumption of a HFD is linked to decreased exercise performance and training efficiency in the offspring. We speculate that this may be due to insufficient muscle energy supply during prolonged exercise training. Further, this compromised exercise performance might increase the risk of obesity development in adult life.

Obesity has a polygenic basis and is modified by nutritional and behavioural factors, including reduced physical activity. Despite the urgent need to develop efficient therapies to fight the rapidly growing obesity epidemic, the knowledge of mechanisms underlying the relationship between early-life nutrition and adult health outcomes remains limited. A growing body of evidence suggests that suboptimal nutritional conditions during pregnancy and lactation can alter the physiology of the offspring and increase their predisposition to many diseases in adult life, such as obesity, impaired glucose tolerance and insulin sensitivity^(^^1^^–^^5^^)^. For skeletal muscle development the fetal stage is of paramount importance since there is no net increase in muscle fibre number after birth^(^^6^^,^^7^^)^. In line with this, disturbed fetal skeletal muscle development was shown to impair skeletal muscle glucose and fatty acid metabolism, predisposing offspring to metabolic diseases later in life^(^^8^^,^^9^^)^.

Offspring of rats fed high-energy diets during gestation and lactation show impaired skeletal muscle development, such as fewer muscle fibres, reduced *ex vivo* muscle power and more intramuscular fat^(^^10^^,^^11^^)^. Thus, one attractive explanation for obesity susceptibility and related disorders is the influence of early-life nutrition on skeletal muscle physiology, especially since muscle makes a major contribution to total energy expenditure^(^^12^^)^. However, it is still unknown to what extent changes in muscle physiology due to prenatal or early postnatal nutrition affect exercise performance in offspring, and how this affects individual obesity susceptibility. Well-directed endurance training triggers the restructuring of skeletal muscle with a switch towards more oxidative fibre types^(^^13^^)^. This implies changes in the metabolic program as well as in structural proteins within myofibres. The restructuring counteracts muscle fatigue by modifying substrate metabolism and contraction properties. However, permanent changes in muscle physiology due to maternal malnutrition could prevent muscle restructuring and impair endurance performance and total energy expenditure. Furthermore, altered muscle physiology due to early overnutrition might have a severe influence on training efficiency which in turn may have an impact on obesity and related diseases in the long term.

Hence the aim of the present study was to scrutinise the interaction between maternal high-fat diet (mHFD) and exercise performance in offspring. In particular, we hypothesise that maternal high fat consumption during gestation and lactation leads in offspring (i) to impaired exercise performance, and (ii) to permanent changes in skeletal muscle physiology.

## Experimental methods

### Animals and diets

All animal studies were conducted in accordance with the Federation of European Laboratory Animal Science Associations (FELASA) guidelines for the care and use of laboratory animals, and all experiments were approved by the ethics committee of the State Agency of Environment, Health and Consumer Protection (State of Brandenburg, Germany, permission no. GZ-23-2347-26-2010). We obtained virgin 8-week-old female (*n* 20) and male (*n* 10) C3H/HeJ mice (Harlan). Animals were randomly allocated to be fed either a semi-synthetic high-fat diet (HFD; 19·7 kJ/g; 40·2 % energy from fat, 23 % energy from protein and 36·8 % energy from carbohydrate; Research Diets Services; Table 1) or a semi-synthetic low-fat diet (LFD; 16·2 kJ/g; 10 % energy from fat, 23·1 % energy from protein, and 66·9 % energy from carbohydrate; Research Diets Services; Table 1)^(^^14^^)^ starting 5 d before mating, at the age of 9 weeks, until weaning. The LFD and HFD are as proposed by BIOCLAIMS and Mitofood consortia and were composed of the same ingredients^(^^14^^)^.

**Table 1.** Composition of the semi-synthetic low-fat diet (LFD) and high-fat diet (HFD)

	LFD	HFD
Composition (g/kg diet)		
Wheat starch	386·5	172·5
Casein	220	267
Maltodextrin	100	100
Sucrose	100	100
Dextrose	50	50
Fat*	43	210
Cellulose	50	50
Mineral mix (AIN-93G-MX)	35	35
Vitamin mix (AIN-93-VX)	10	10
l-Cysteine	3	3
Choline bitartrate	2·5	2·5
Total energy (kJ/g diet)	16·2	19·7
% Energy from carbohydrates	66·9	36·8
% Energy from protein	23·1	23·0
% Energy from fat	10·0	40·2

* Combination of sunflower-seed oil (70 %), coconut oil (18 %) and flaxseed oil (12 %).

At mating the average weights of females were 22·41 (se 1·09) and 23·75 (se 1·34) g, respectively, for the LFD and HFD treatments. Two females were introduced to one male for 1 week and subsequently individually caged. After delivery litter size was adjusted to between five and seven pups. Post-weaning male offspring (21 d), of both maternal diet groups (maternal LFD (mLFD) or mHFD), were housed individually and received the LFD and water *ad libitum* throughout the rest of the study. All animals were kept at 22°C with a 12 h light–12 h dark cycle. Body mass of all experimental mice was measured twice a week to the nearest 0·1 g using an electronic balance. Food was removed 2 h before animals were anaesthetised and killed by cervical dislocation in week 12, 24 h after an exercise capacity test. Tissues were removed, snap-frozen in liquid N_2_ and stored at –80°C until analyses.

### Body composition

At the age of 7 and 12 weeks body composition (fat mass and lean mass) were assessed non-invasively as described previously^(^^15^^,^^16^^)^ using quantitative magnetic resonance (QMR). Lean body mass was calculated by subtracting body fat mass obtained by QMR from body mass.

### Voluntary exercise training

At the age of 7 weeks half of the mLFD (*n* 20) and the mHFD (*n* 21) groups were given access to a running wheel (RW) (TSE Systems) for 28 d as a voluntary exercise training opportunity, resulting in four groups of offspring: mLFD + RW, mLFD–RW, mHFD + RW and mHFD–RW. Daily wheel-running and spontaneous activity (using IR motion detectors; TSE Systems) were monitored for 28 d. RW usage occurred only during night-time (lights off period). Therefore, only night-time RW usage and spontaneous activity is presented. A freely accessible RW for exercise training rather than forced exercise training was chosen because it reflects more the modern lifestyle in our society. Furthermore, we used a voluntary running model because mice run in a self-controlled physically capable manner in contrast to a forced exercise protocol which potentially introduces confounding physiological and psychological variables^(^^17^^)^.

### Exercise capacity and training efficiency

Exercise capacity was determined before (at the day of RW access) and after (24 h before tissue collection) voluntary exercise training. Mice were placed on a six-lane treadmill (Columbus Instruments). Mice were given the opportunity to adjust to the treadmill 1 d before determination of the exercise capacity. The starting speed of 12 cm/s was increased every 5 min by 4 cm/s until a final speed of 48 cm/s was reached. The mice continued to run at 48 cm/s until exhaustion. Continuous contact with the electrical shock grid at the end of each running lane for more than five successive seconds was defined as exhaustion. Training efficiency of each individual mouse was calculated as the difference in exercise performance (distance run) between the first and second measurement of exercise capacity.

### Energy expenditure

Before (week 7) and after (week 12) voluntary exercise training energy expenditure and respiratory quotient (RQ) were determined by indirect calorimetry over a period of 23 h, according to published protocols^(^^15^^,^^18^^)^. Total energy expenditure was normalised to 24 h. Resting energy expenditure (REE) and RQ were calculated as described^(^^19^^)^.

### Analysis of mRNA expression

Muscle tissue samples (*M. quadriceps femoris, M. gastrocnemius, M. tibialis and M. soleus*) were removed and immediately frozen in liquid N_2_ for real-time PCR assays. Total RNA from homogenised muscle tissues was extracted using a single-step acid phenol–guanidine method as described before^(^^20^^)^ with modifications described by Weber *et al.*^(^^21^^)^. Quality and integrity of the RNA were checked by calculating the A260:A280 ratio and by agarose gel electrophoresis. A sample of 1 µg of total RNA was treated using the Turbo DNA-free Kit (Ambion) and reverse-transcribed using the RevertAid H Minus First Strand complementary DNA Synthesis Kit (Fermentas). Quantitative real-time PCR was performed on the Applied Biosystems 7900 HT Fast Real-Time PCR System using Taqman SYBR® Green Master Mix (Applied Biosystems) with the standard PCR protocol as recommended by the manufacturer and about 5 ng cDNA. Dissociation curve analysis was performed after the amplification step to verify the presence of only a single PCR product. All primers were designed from sequences obtained from GenBank: sarcoplasmic/endoplasmic reticulum Ca ATPase 1 and 2 (Serca1, Serca2), oestrogen-related receptor-γ (Esrrg), PPAR-γ, coactivator 1α (PGC1α), carnitine O-palmitoyltransferase 1b (Cpt1b), cluster of differentiation 36 (CD36), fatty acid synthase (FAS), GLUT1, GLUT4, 18S rRNA and β-2-microglobulin (B2m). The last two were evaluated as reference (housekeeping) genes and showed no changes in expression levels between groups. Gene expression was calculated according to Pfaffl^(^^22^^)^ as ΔCT (cycle threshold), normalised with 18S rRNA or B2m, and expressed relative to the mLFD–RW group.

### TAG and glycogen analyses

For TAG analysis, liver tissue (100 mg) and muscle tissue (40–50 mg) were homogenised in 50 volumes or for muscle tissue in 20 volumes of 10 mm-sodium phosphate buffer, pH 7·4, containing 1 mm-EDTA and 1 % polyoxyethylene-10-tridecyl ether using an Ultra-Turrax (IKA-Werke). Samples were centrifuged for 15 min at 23 100 ***g***. The supernatant fraction was incubated at 70°C for 5 min and centrifuged again. TAG (triglyceride reagent; Sigma) and protein contents (detergent compatible protein assay; Bio-Rad) were analysed in triplicates from the supernatant fraction. For glycogen analysis, 50 mg of ground liver or muscle tissue were homogenised in 1 ml of 0·1 m-NaOH using an Ultra-Turrax. The homogenate was incubated at 70°C for 45 min and centrifuged for 10 min at 48°C and 12 400 ***g***. Glycogen (Starch Kit; R-Biopharm) and protein contents (detergent-compatible protein assay) were analysed in triplicates from the supernatant fraction.

### Citrate synthase assay

Muscle tissue was homogenised in nineteen volumes of 50 mm-Tris/HCl, 1 mm-EDTA and 0·1 % Triton X-100, pH 7·4. Assays were performed in triplicates at 37°C. Enzyme activities were determined according to published protocols^(^^23^^)^.

### Immunological detection

Protein extraction from *M. quadriceps*, SDS-PAGE and incubation of different antibodies, as well as chemiluminescence detection and quantification of protein bands were performed as described before^(^^24^^)^. CD36 (R&D Systems) and α-tubulin (Sigma-Aldrich) were used as primary antibodies and either anti-rat (R&D Systems) or anti-mouse IgG (Cell Signaling Technology) horseradish peroxidase-conjugated secondary antibodies were used.

### Statistical analysis

All data were analysed using SPSS (version 16; SPSS, Inc.) or Graphpad Prism (version 5; GraphPad Software, Inc.). Data are reported as mean values with their standard errors. All data were tested for normality and homogeneity of variance using Levene's test. If a dataset failed Levene's test the data were ln transformed. We compared the mLFD with the mHFD group before voluntary training by *t* tests for independent samples, and body mass data were analysed by repeated-measures ANOVA. Different RW treatment groups and maternal diet groups were compared by two-way ANOVA followed by Bonferroni *post hoc* tests. Additionally, body mass data were analysed by repeated-measures two-way ANOVA followed by Bonferroni *post hoc* tests. Significance was considered as *P* < 0·05.

## Results

### Early effects of maternal diet before voluntary training

**Fig. 1.**
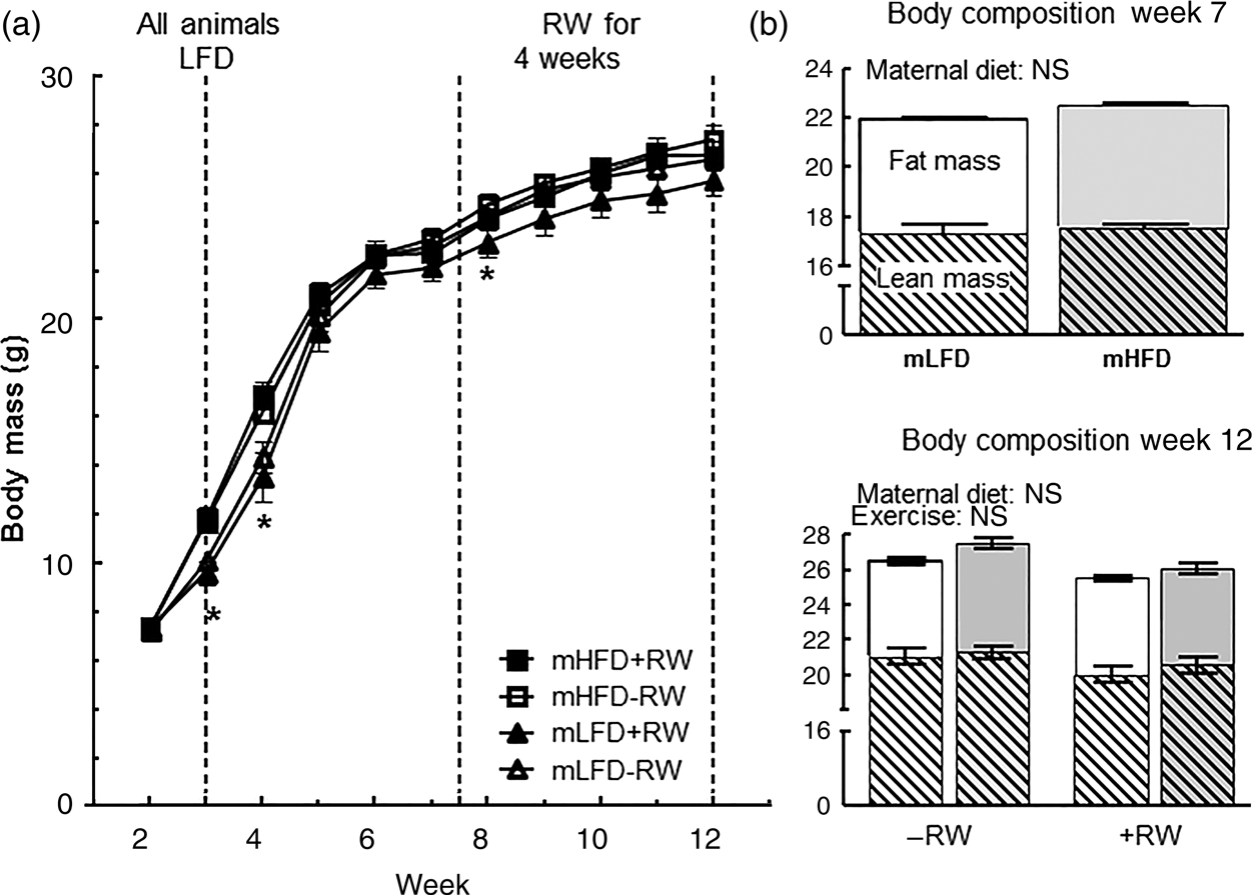
Influence of maternal high-fat diet (mHFD) on offspring body mass and composition. (a) Body mass development of male mice offspring from two dietary groups with or without running wheel (RW) access (*n* 10–11 per group). -■-, mHFD with RW access (mHFD + RW); -□-, mHFD without RW access (mHFD–RW); -▴-, maternal low-fat diet (mLFD) with RW access (mLFD + RW); -▵-, mLFD without RW access (mLFD–RW). Values are means, with standard errors represented by vertical bars. Body mass data were analysed by repeated-measures ANOVA. * Mean values were significantly different between the groups (*P* < 0·05). (b) Body composition of male mice offspring from two dietary groups with (RW+) or without (RW–) RW access (*n* 10–11 per group). □, mLFD; 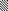, mHFD. Values are means, with standard errors represented by vertical bars. Body composition at week 7 was compared by Student's *t* test and at week 12 by two-way ANOVA. Main effects for maternal diet and/or exercise are stated.

**Fig. 2.**
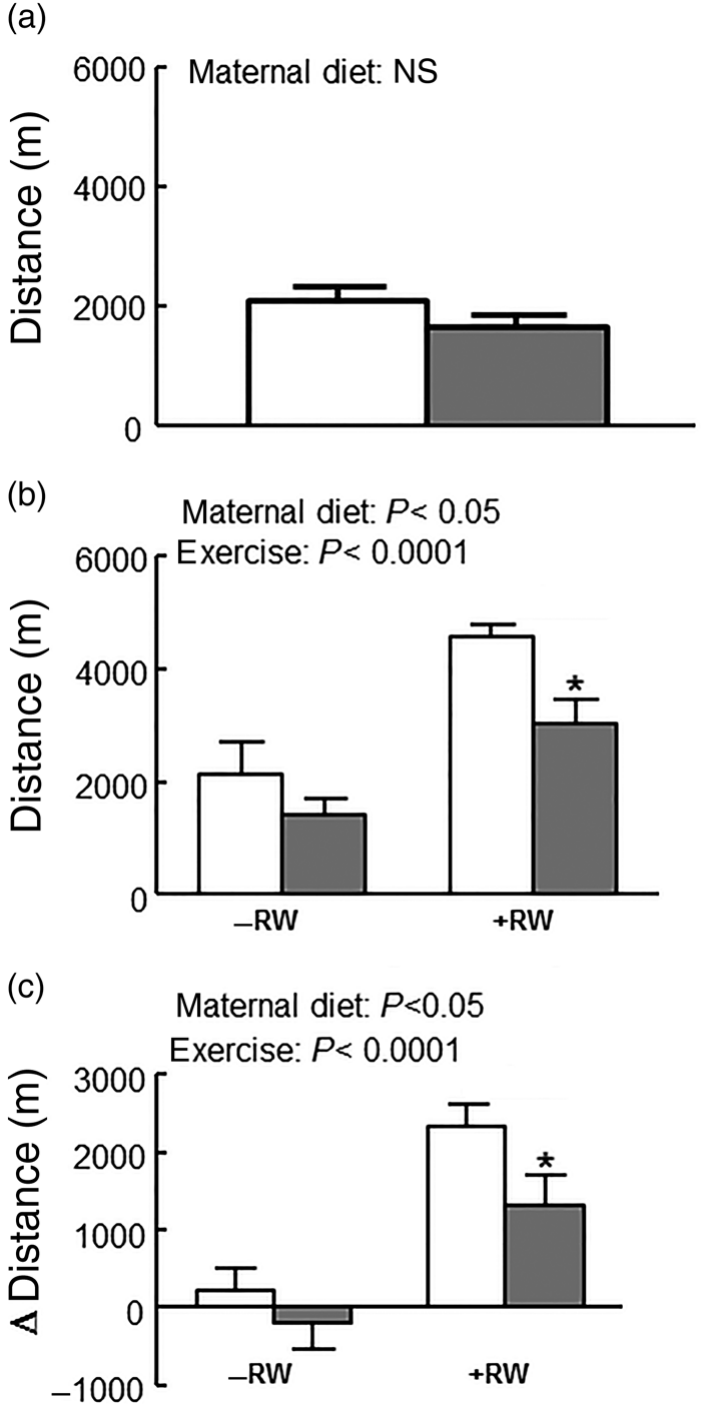
Influence of maternal high-fat diet (mHFD; 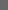 ) on offspring exercise capacity and training response. (a) Exercise test before voluntary training (b), after 4 weeks of voluntary training and (c) training efficiency of male mice offspring from two dietary groups with running wheel (RW) access (RW+) or without RW access (RW–) (*n* 10–11 per group). □, Maternal low-fat diet (mLFD). Exercise capacity was measured using an incremental treadmill running test to exhaustion protocol. Training efficiency was calculated as the difference in exercise capacity before and after voluntary exercise training for each mouse. Values are means, with standard errors represented by vertical bars. Exercise data were compared by Student's *t* test before RW access and by two-way ANOVA after RW access. Main effects for maternal diet and/or exercise are stated. * Mean value was significantly different from that of the mLFD mice within the same exercise group (*P* < 0·05).

As shown in Fig. 1, mHFD consumption led to about 30 % increased body mass in offspring at weeks 3 and 4 after birth. After week 4 these differences disappeared. Also there were no differences in lean and fat mass measured at week 7. REE was about 10 % higher in the mLFD group than in the mHFD group (*P* < 0·05) before exercise training but these differences disappeared in week 12 when also total energy expenditure over 24 h did not differ between groups (Table 2). The resting RQ during rest (REE RQ) was similar among groups (Table 2). Also both groups consumed similar quantities of food throughout the experiment (Table 2). To test exercise endurance directly, untrained mice of both groups were challenged with a single run-to-exhaustion trial. Individual exercise capacity did not differ between the groups (Fig. 2(a)).

**Table 2.** Influence of maternal high-fat diet (mHFD) on offspring energy metabolism and activity (Mean values with their standard errors)

	No exercise (–RW)				Exercise ( + RW)					
	mLFD		mHFD		mLFD		mHFD		ANOVA: *P*	
	Mean	se	Mean	se	Mean	se	Mean	se	Maternal diet	Exercise
Before exercise (week 6)										
Food intake (kJ/animal per d)	43·81	2·7	50·41	2·23					NS*	
REE (kJ/h)	36·17	0·80	32·83	1·15					<0·05*	
REE RQ	0·77	0·01	0·76	0·01					NS*	
After exercise (week 12)										
Food intake (kJ/animal per d)	54·35	4·59	62·0	2·20	63·55	2·20	59·0	5·08	NS	NS
TEE (kJ/h)	57·51	2·63	59·39	1·14	59·32	0·81	58·42	1·37	NS	NS
REE (kJ/h)	38·55	1·26	41·22	1·29	39·26	0·95	38·32	1·25	NS	NS
REE RQ	0·798	0·01	0·81	0·02	0·764	0·008	0·750	0·007	NS	<0·0001
RW usage at night (rotations/night)	–		–		15730	1778	14020	1485	NS	–
Activity at night (counts/night per 1000)	42·79	6·29	46·45	3·76	65·06	64·24	70·26	4·50	NS	<0·0001

RW, running wheel; mLFD, maternal low-fat diet; REE, resting energy expenditure; RQ, respiratory quotient; TEE, total energy expenditure.

* *t* test.

### Voluntary training

To examine if early HFD consumption leads to impaired exercise performance, half of the mice of both groups had access to a RW for 4 weeks. Daily (i.e. night-time) RW usage did not differ between the maternal diet groups (Table 2). However, animals with access to a RW were 1·5 times more active during night than animals without RW access which was independent of the maternal diet (*P* < 0·0001; Table 2). Body mass did not differ among the groups during 4 weeks of voluntary training (Fig. 1).

Voluntary exercise training doubled the exercise capacity of both diet groups compared with sedentary mice (*P* < 0·0001; Fig. 2(b)). Furthermore, mHFD consumption led to a significantly reduced exercise performance, which was about 50 % reduced in the mHFD group compared with the mLFD group with RW access (*P* < 0·05; Fig. 2(b)). Also training efficiency was significantly reduced by maternal high fat consumption (*P* < 0·05), the efficiency being twice as high in mLFD as in mHFD mice in the groups with RW access (Fig. 2(c)). Body mass as well as fat and lean mass did not differ among the groups (Fig. 1). However, REE RQ, a measure of metabolic substrate use, was significantly reduced in the groups with RW access, indicating preferential oxidation of fat as a substrate for energy metabolism (Table 2). Total energy expenditure and REE were similar for all the four treatment groups. Also, mice of all groups consumed similar quantities of food (Table 2).

**Fig. 3.**
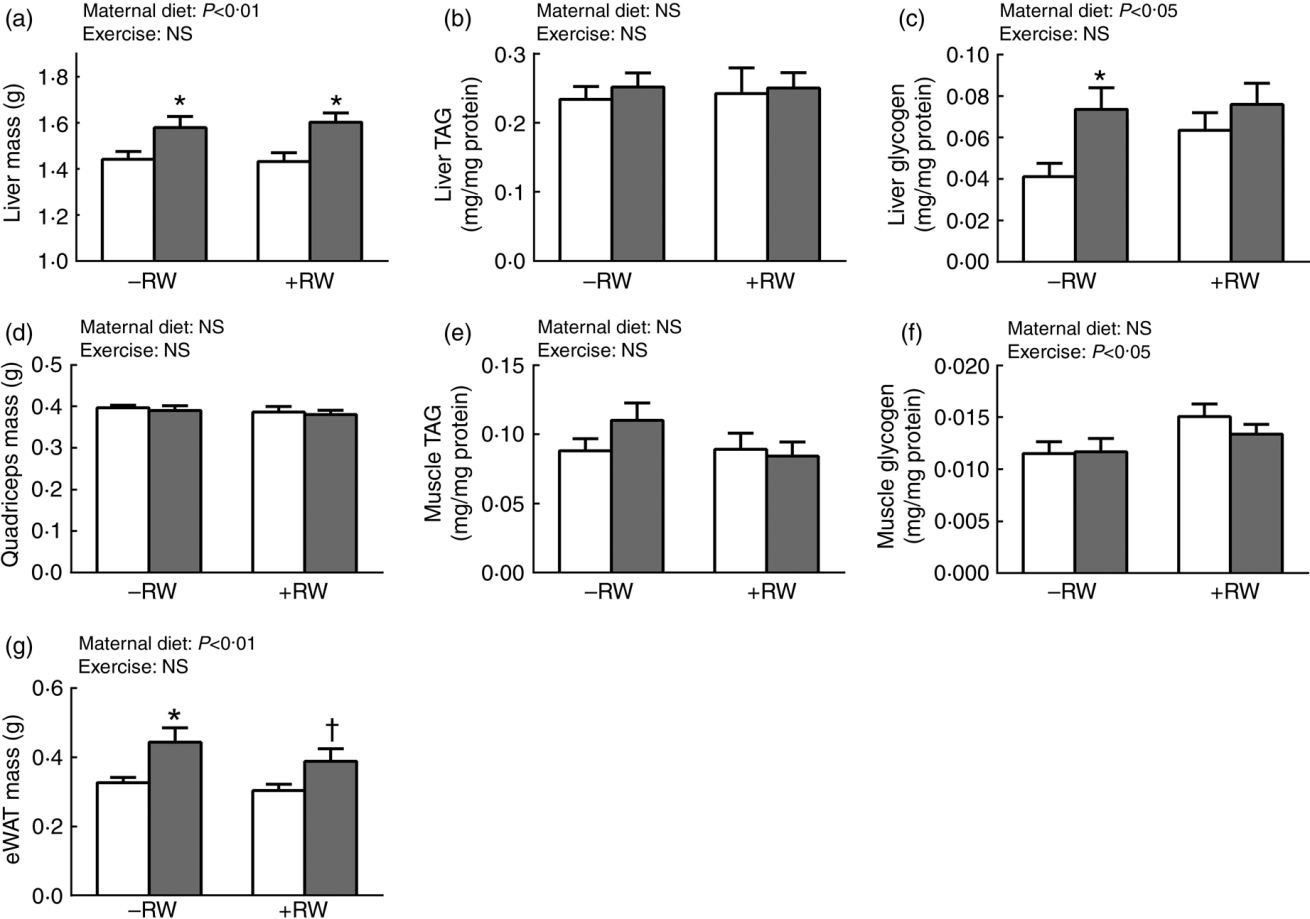
Influence of maternal high-fat diet (mHFD; 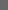 ) on offspring tissue mass, and TAG and glycogen content. Liver mass (a), liver TAG content (b), liver glycogen content (c), muscle mass (d), muscle TAG content (e), muscle glycogen content (f) and epididymal white adipose tissue (eWAT) mass of maternal low-fat diet (mLFD; □) and mHFD male offspring. Half of both groups had access to a running wheel (RW+) for 4 weeks; half were without access (RW–). Values are means (*n* 10–11 per group), with standard errors represented by vertical bars. Data were compared by two-way ANOVA. Main effects for maternal diet and/or exercise are stated. * Mean value was significantly different from that of the mLFD mice within the same exercise group (*P* < 0·05). † Mean value was marginally significantly different from that of the mLFD mice within the same exercise group (*P* = 0·06).

Livers of mHFD offspring were 10 % heavier compared with liver mass of the mLFD group (*P* < 0·001; Fig. 3(a)). Epididymal fat mass (epididymal white adipose tissue; eWAT) mass was 25 % increased in the mHFD group (*P* < 0·01; Fig. 3(g)), but *M. quadriceps* mass was unchanged by treatments (Fig. 3(d)). Body mass was not different between the groups; therefore these effects were very much the same for relative tissue weights (data not shown). Differences in liver mass were associated with differences in glycogen content (*P* < 0·05; Fig. 3(c)), whereas TAG content was similar among the groups (Fig. 3(b)). Voluntary exercise training increased the glycogen content in *M. quadriceps* of both treatment groups by about 20 % (*P* < 0·05; Fig. 3(f)) but TAG content was unchanged by treatments (Fig. 3(e)).

**Fig. 4.**
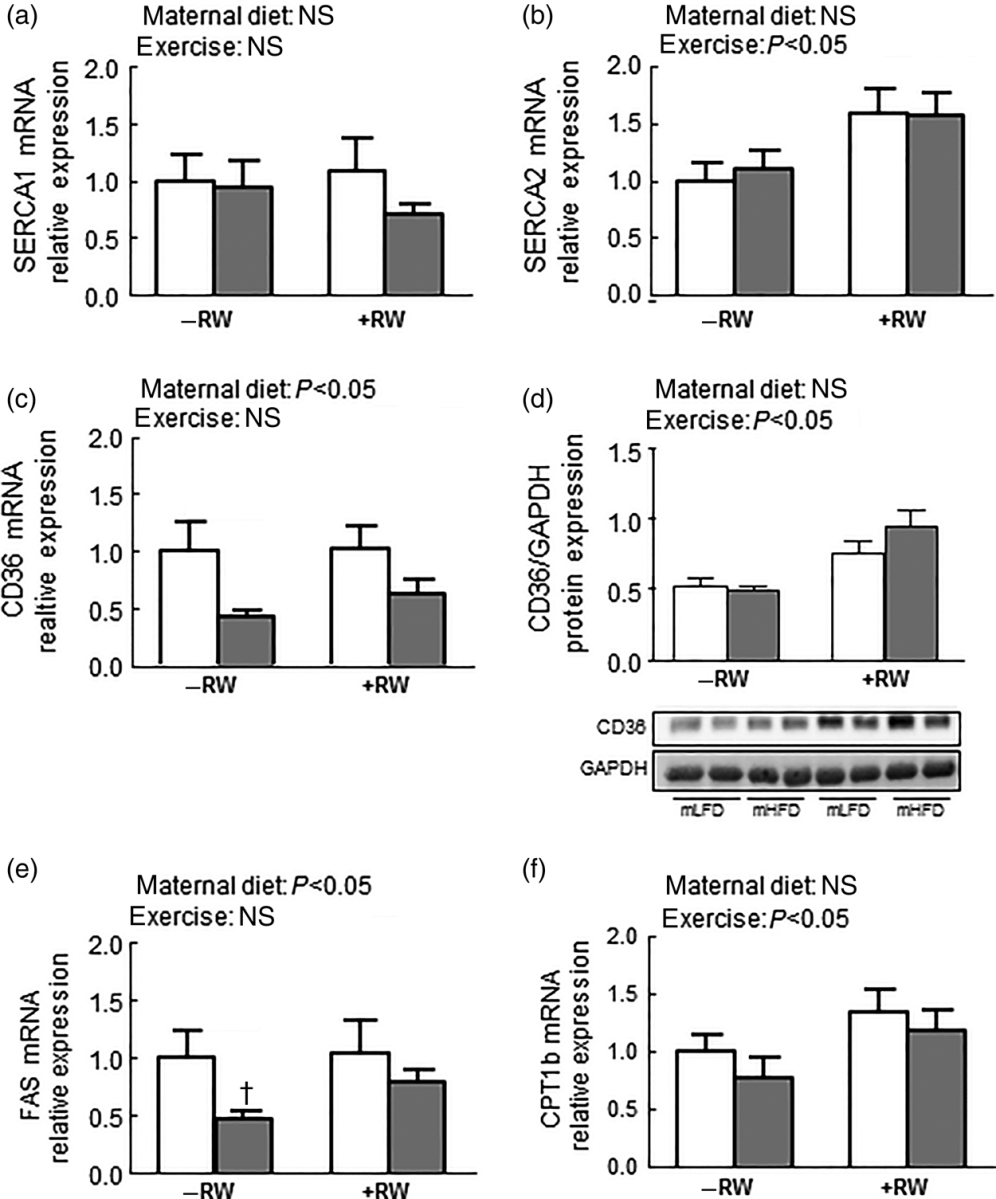
Influence of maternal high-fat diet (mHFD; 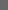 ) on offspring muscle gene and protein expression. Gene expression of sarcoplasmic/endoplasmic reticulum Ca ATPase 1 (SERCA1) (a), SERCA2 (b) and cluster of differentiation 36 (CD36) (c), protein expression of CD36 relative to glyceraldehyde 3-phosphate dehydrogenase (GAPDH) (d), and gene expression of fatty acid synthase (FAS) (e) and carnitine O-palmitoyltransferase 1b (CPT1b) (f) in skeletal muscle of maternal low-fat diet (mLFD; □) and mHFD male offspring. Half of both groups had access to a running wheel (RW+) for 4 weeks; half were without access (RW–). Values are means (*n* 10–11 per group), with standard errors represented by vertical bars. Data were compared by two-way ANOVA. Main effects for maternal diet and/or exercise are stated. * Mean value was significantly different from that of the mLFD mice within the same exercise group (*P* < 0·05). † Mean value was marginally significantly different from that of the mLFD mice within the same exercise group (*P* = 0·05).

Isolated quadriceps muscle (a mixed fibre-type muscle important for exercise capacity) exhibited no differences in the expression of Serca1, a marker for fast type II muscle fibres. On the other hand, Serca2, an indicator of slow type I muscle fibres, was about 50 % increased in both exercise groups (Serca2: *P* < 0·01; Fig. 4(a) and 4(b)).

mRNA levels of CD36 and FAS, important for fatty acid metabolism, were significantly decreased due to mHFD consumption (Fig. 4(c) and 4(e)), an effect that was more pronounced in the groups with no RW access. Although RW access did not influence CD36 gene transcription levels, protein expression levels of CD36 as well as Cpt1b gene expression levels, important for fatty acid oxidation, were increased in both groups with access to a RW (Fig. 3(d) and 3(f)). The expression pattern of metabolic genes was very similar in the other muscle types isolated (data not shown).

GLUT1 mRNA levels, important for basal glucose uptake, were about 40 % decreased due to mHFD consumption whereas GLUT4 mRNA remained unchanged with treatments (GLUT1: *P* < 0·05; Table 3). We could not detect any changes in PPAR-γ, coactivator 1α (PGC1α) gene expression and citrate synthase activity (Table 3). Also, protein expression of complexes 1 to 5 of the mitochondrial respiratory chain was not different between the groups (data not shown). However, mHFD consumption significantly increased the expression of Esrrg by over 60 % (Table 3).

**Table 3.** Effect of maternal high-fat diet (mHFD) on offspring muscle gene expression and physiology (week 12) (Mean values with their standard errors)

	No exercise (–RW)				Exercise ( + RW)					
	mLFD		mHFD		mLFD		mHFD		ANOVA: *P*	
	Mean	se	Mean	se	Mean	se	Mean	se	Maternal diet	Exercise
Mitochondrial biogenesis and activity										
PGC1α mRNA	0·999	0·10	1·258	0·22	1·19	0·22	1·154	0·15	NS	NS
Citrate synthase activity	20·77	0·65	20·64	1·17	22·84	0·61	22·01	1·44	NS	NS
Esrrg mRNA	1·00	0·15	1·64	0·14	1·24	0·13	1·69	0·18	<0·001	NS
Glucose uptake										
GLUT1 mRNA	1·00	0·21	0·58	0·04	0·74	0·10	0·61	0·07	<0·05	NS
GLUT4 mRNA	1·00	0·13	1·10	0·10	1·34	0·18	1·28	0·12	NS	NS

RW, running wheel; mLFD, maternal low-fat diet; PGC1α, PPAR-γ, coactivator 1α; Essrg, oestrogen-related receptor-γ.

## Discussion

Here we show that mHFD consumption during pregnancy and lactation leads to a marked reduction in exercise performance and training efficiency in young adult male offspring. In addition, maternal high fat feeding alters gene expression important for lipid metabolism in skeletal muscle of offspring in a manner indicative of decreased fat synthesis. Therefore, we speculate that the effect of mHFD consumption on exercise capacity in voluntary-trained offspring might be due to an insufficient energy supply during prolonged exercise training.

It is known that there is a strong association between low aerobic exercise capacity and metabolic dysfunction^(^^25^^,^^26^^)^. At present, the only effective treatment for the improvement of exercise capacity is exercise training. However, exercise training in human subjects is known to result in significant variations in the ability to improve exercise capacity including even exercise-resistant individuals^(^^27^^–^^29^^)^. One explanation why some individuals are exercise resistant might be mHFD consumption during pregnancy and lactation. The present results clearly show a reduced training capacity of offspring exposed to perinatal high fat feeding (mHFD). From weaning onwards all offspring received the same LFD, which clearly points to a perinatal programming of muscle training capacity.

Endurance training triggers a restructuring of skeletal muscle with a switch towards more oxidative type I fibres^(^^13^^)^. This is in line with our observation of an increased gene expression of Serca2, a marker for type I muscle fibres^(^^30^^)^ in both groups with RW access. mHFD consumption on the other hand did not influence the fibre type distribution in *M. quadriceps*, suggesting that fibre type changes are independent of perinatal nutrition. During exercise training the main metabolic fuel sources for ATP generation in muscle cells are glucose and – if energetic demands are submaximal but prolonged – fatty acids^(^^31^^)^. Hence, as expected, glycogen storage within the muscle as well as protein expression of the fatty acid transporter CD36 and mRNA levels of CPT1b were increased in both groups with RW access reflecting the elevated demands in energy supply during exercise training. Substrate oxidation occurs in the mitochondria, and citrate synthase activity is commonly used as a marker of mitochondrial density^(^^32^^)^. Our observed lack of a training effect on muscle citrate synthase activity might be due to an insufficient training volume or training time span. However, the present results clearly show training effects of 4 weeks of voluntary training via a RW as evident from significant effects on exercise capacity and muscle physiology as well as markers of fibre type (Serca2) and of fatty acid metabolism (CD36 protein and CPT1b gene expression). The reduction in exercise performance observed by mHFD exposure is most likely not due to an altered skeletal muscle mitochondrial integrity because markers for mitochondrial activity and density (citrate synthase activity and content of respiratory chain proteins) were not different between the groups.

Interestingly, perinatal HFD exposure-affected targets were distinct from those affected by exercise, such as increased liver and eWAT mass, and increased hepatic glycogen content in mHFD offspring. Also mHFD targets in skeletal muscle itself were different from exercise-affected targets. Notably, lipogenesis (FAS gene expression) and basal glucose uptake (GLUT1 gene expression) in muscle were significantly reduced by mHFD feeding in the absence of any exercise effects. Altogether, these observations suggest that substrate availability following energy allocation differs between the maternal high and low fat-exposed offspring. These findings suggest a predisposition of mHFD offspring to decreased fatty acid availability during endurance training which might lead to decreased exercise performance. Regular aerobic exercise has been linked to decreased body mass, improved glucose homeostasis and enhanced insulin sensitivity in human subjects and rodents^(^^33^^,^^34^^)^. Hence, reduced exercise ability in offspring, due to mHFD consumption, might have long-term consequences on the development of obesity and related diseases later in life.

The decreased exercise performance of the mHFD + RW group was not accompanied by differences in body mass or energy expenditure. However, RW exposure reduced eWAT mass of the mHFD-exposed mice, indicating that voluntary running reversed the increased eWAT mass caused by the perinatal HFD consumption. Recently, Ashino *et al.* reported that an increased supply of nutrients before birth led to a decreased phosphorylation of hormone-sensitive lipase in eWAT which resulted in increased eWAT depots later in life^(^^35^^)^. Beside effects on eWAT, we observed an increased liver weight in mHFD offspring linked to an increased hepatic glycogen accumulation. A larger reserve of hepatic glycogen may reflect increased gluconeogenesis. Franco *et al.* showed in rats that a perinatal mHFD led to increased hepatic glycogen storage associated with a decrease of β2-adrenoreceptors in offspring at weaning^(^^36^^)^, which is in line with our findings. On the other hand, they fed female rats the HFD over 8 weeks before mating which led to hyperglycaemia in offspring at weaning whereas we did not find any differences in blood glucose in offspring (data not shown). It has been discussed that mHFD consumption during the gestational period is associated with an elevated gluconeogenic capacity in offspring due to epigenetic modification of the phosphoenolpyruvate carboxykinase 1 gene^(^^37^^)^. In contrast to Ashino *et al.*^(^^35^^)^ we could not detect any differences in hepatic TAG content in mHFD offspring. One explanation could be that in the present study dams of both diet groups showed no differences in body mass throughout lactation until 7 d post-partum. It was suggested that maternal lipid transfer might be associated with permanent metabolic changes in fetal liver, such as reduced TAG export and decreased fatty acid oxidation in mHFD offspring^(^^35^^,^^38^^)^. Therefore we did not expect any differences in hepatic lipid content in the mHFD group.

mHFD consumption affected muscle Esrrg gene expression in offspring. Unlike Esrra and β, Esrrg is more selectively expressed in metabolically active and highly vascularised tissues such as skeletal muscle, heart, kidney and brain^(^^39^^,^^40^^)^. It has been reported to play an important role in the shift toward slow twitch muscle type and therefore in the increased capacity for endurance exercise by controlling the expression of key genes in angiogenic, myofibrillar and Ca-handling pathways in skeletal muscle^(^^41^^)^. Since Esrrg is also important in the control of energy metabolism, such as the citric acid cycle and fatty acid oxidation^(^^40^^,^^42^^)^, we speculate that Esrrg is important for the regulation of glycolysis and fatty acid oxidation in the muscle of mHFD offspring. However, it does not seem to be involved in fibre type switch since muscle Esrrg gene expression was not regulated in both exercised groups.

It should be pointed out that we used a voluntary exercise regimen rather than forced exercise training. Forced training protocols, such as swimming or treadmill running, use aversive stimuli, such as the possibility of drowning or electrical shocks. The advantage of forced exercise training is its reproducibility in distances and speeds. However, the conditions are non-physiological since training sessions are stressful and normally performed in daylight hours, contrary to the nocturnal activity in mice. It has been shown that stress levels induced by forced exercise can affect brain function and exacerbate inflammation^(^^43^^,^^44^^)^. On the other hand, voluntary-running activity occurs in a non-stressed environment and without violating the diurnal rhythm of mice. Also, we monitored voluntary wheel running activity to be assured that both RW groups used the RW with the same intensity and duration.

In conclusion, we have demonstrated for the first time that perinatal exposure to mHFD consumption results in decreased exercise performance and training efficiency in offspring. The exact molecular mechanisms still need to be evaluated, but our data suggest the involvement of direct effects on muscle function linked to a compromise of muscle energy supply. Interestingly, these changes occurred without maternal obesity and may contribute, as priming factors, to the development of obesity later in life. It will be of interest to elucidate if the maternal impact on training efficiency in offspring is linked to epigenetic modifications of genes important for glucose and lipid metabolism in skeletal muscle.
